# 

**Published:** 1887-12

**Authors:** 


					To Readers and Correspondents!
Communications intended for publication, and exchange journals and
papers should be addressed to the Editor of the American Journal of
Dental Science, 845 N. Eutaw St., Baltimore, Md. All letters relating
to the business of the Journal, or containing cash remittances, to be
directed to Messrs. Snowden & Cowman. Articles for publication should
be received by the'editor at least three weeks previous to the first month
on which the number in which it is designed for them to appear, is issued.
The American Journal of Dental Science will contain fifty
pages of reading matter in each number, making a volume of about
Six Hundred pages,
EXCLUSIVE OF ADVERTISEMENTS.

				

